# Pooled Prevalence of Long COVID-19 Symptoms at 12 Months and Above Follow-Up Period: A Systematic Review and Meta-Analysis

**DOI:** 10.7759/cureus.36325

**Published:** 2023-03-18

**Authors:** Shiv K Mudgal, Rakhi Gaur, Satyaveer Rulaniya, Latha T, Rajat Agarwal, Subodh Kumar, Saurabh Varshney, Suresh Sharma, Sudip Bhattacharya, Vasantha Kalyani

**Affiliations:** 1 College of Nursing, All India Institute of Medical Sciences Deoghar, Deoghar, IND; 2 College of Nursing, All India Institute of Medical Sciences Jodhpur, Jodhpur, IND; 3 College of Nursing, All India Institute of Medical Sciences Kalyani, West Bengal, IND; 4 Department of Cardiothoracic Surgery, All India Institute of Medical Sciences Deoghar, Deoghar, IND; 5 Department of Pharmacology, All India Institute of Medical Sciences Deoghar, Deoghar, IND; 6 Department of Otolaryngology, All India Institute of Medical Sciences Deoghar, Deoghar, IND; 7 Department of Community Medicine, All India Institute of Medical Sciences Deoghar, Deoghar, IND

**Keywords:** a systematic review, symptoms, prevalence, meta-analysis, long term covid-19

## Abstract

Current data suggests that coronavirus disease 2019 (COVID-19) survivors experience long-lasting problems. It is not yet understood how long these symptoms last. The goal of this study was to compile all the data that was currently available to evaluate COVID-19's long-term effects at 12 months and above. We looked for studies published by December 15, 2022, in PubMed and Embase that discussed follow-up findings for COVID-19 survivors who had been alive for at least a year. A random-effect model was carried out to determine the combined prevalence of different long-COVID symptoms. The Joanna Briggs Institute tool was used to assess the risk of bias for the included studies, and the I2 statistics were used to evaluate the heterogeneity. After reviewing 3,209 studies, 46 were deemed admissible, with an aggregate COVID-19 population of 17976. At 12 months and above, 57% of patients reported a minimum of one symptom, and the five most prevalent symptoms were: dyspnea on exertion (34%, 95% CI 0.2; 0.94); difficulty in concentration (32%, 95% CI 0.16; 0.52); fatigue (31%, 95% CI 0.22; 0.40); frailty (31%, 95% CI 0.06; 0.78); and arthromyalgia (28%, 95% CI 0.09; 0.6). The findings of the present study showed that at 12 months and beyond, a sizable fraction of COVID-19 survivors still have lasting symptoms that impair several body systems. Long-COVID patients require an urgent understanding of pathophysiological processes and the development of tailored treatments.

## Introduction and background

Presently, the biggest threat to global public health is the coronavirus disease 2019 (COVID-19) pandemic. The World Health Organization (WHO) recorded approximately 668 million COVID-19 confirmed cases as of January 8, 2023, with over six million fatalities [1].

After recovering from an acute COVID-19 infection, a sizable proportion of patients report continuing symptoms that interfere with daily activities after the initial acute stage. A condition referred to as "Long COVID-19," "post-acute COVID-19," "persistent COVID-19 symptoms," "chronic COVID-19," "long-term sequelae," or "long-haulers" has been used to describe these post-COVID-19 patients. More recently, "post-acute sequelae of COVID-19" or "post-acute COVID-19 Syndrome (PACS)" has also been used to describe these individuals [2,3]. The World Health Organization (WHO) published a clinical case description for the post-COVID-19 condition using a Delphi consensus method on October 6, 2021: “the condition affects people who have a history of probable or confirmed SARS-CoV-2 infection, typically three months after the onset, and has symptoms that last for at least two months and cannot be accounted for by any other diagnosis.” Another review suggested long COVID-19 when symptoms last longer than four weeks after onset and/or problems develop months or years later [3,4].

Evidence is mounting that COVID-19 survivors may continue to have symptoms that involve several organ systems following the acute stage of the illness (also known as long-COVID) [5]. In multiple earlier systematic reviews and meta-analyses on long-COVID-19 infections, the prevalence of short- and medium-term persistent cardiac, respiratory, neurological, integumentary, musculoskeletal, and gastrointestinal symptoms was evaluated [6-9]. A meta-analysis [7] of 33 articles reported that 63.2%, 71.9%, and 45.9% of COVID-19 survivors had a minimum of one persistent symptom 30, 60, and 90 days after hospitalization or onset, and fatigue, difficulty breathing, cough, anosmia, ageusia, and joint pain are the most common symptoms. The most frequently reported symptoms, according to another meta-analysis [8] of 39 studies with seven months of follow-up, were weakness, fatigue, impaired focus, and shortness of breath. Another meta-analysis [6] of 15 publications found that COVID-19 survivors experienced over 50 persisting symptoms between 14 and 110 days following infection.

For longer-term persistent symptoms of COVID-19, it is unclear how long these various groups of symptoms will last. In view of the growing body of information on longer-duration follow-up of COVID-19 survivors, it is imperative to determine whether the pattern of persistent symptoms is different from the previously documented short- or medium-term symptoms. To offer a better picture of the long-term effects of COVID-19 based on scientific data, we sought to synthesize the evidence by pooling related studies related to the long-term effects of COVID-19 at 12 months and beyond. It will assist with the management and monitoring of COVID-19 survivors as well as the development of public health policies for medical services.

## Review

Methods

The Preferred Reporting Items for Systematic Reviews and Meta-analyses statement was followed by this systematic review and meta-analysis [10]. The registration number for this study with PROSPERO is CRD42022355069.

Search strategy

We carried out a thorough search of the PubMed and Embase databases for articles that presented results for people who survived COVID-19 at 12-month or longer follow-ups, published after January 31, 2021, and in English only. The following search keywords yielded results: “(COVID-19 OR SARS-CoV-2 OR coronavirus OR long COVID-19 OR post-COVID-19)” AND “(long-term effect OR sequelae OR consequences)” AND “(cohort OR follow-up OR retrospective OR prospective)”. The searches merged free-text words with Medical Subject Headings (MeSH) phrases.

Study selection

The following criteria were met for articles to be considered for inclusion: (a) Cohort, cross-sectional, or case-control peer-reviewed studies reporting the prevalence of long-term symptoms among COVID-19 survivors; (b) articles published in the English language; (c) including a minimum of 50 COVID-19 survivors; (d) at least a follow-up period of 12 months after symptom onset; (e) patient inclusion of only those with laboratory-confirmed COVID-19. The following studies were left out: (a) studies where SARS-CoV-2 infection was not used as the exposure; (b) studies that did not have enough data to figure out what the long-term COVID-19 symptoms are; (c) duplicate studies or studies with the same participants; (d) editorials, reviews, case reports/series, conference papers, secondary analysis, or animal studies; and (e) qualitative designs.

Using the previously stated specific eligibility criteria, four reviewers independently looked at the titles along with the abstracts of the articles in pairs. Following that, each article was subjected to a full-text review to ensure that it met the eligibility criteria. The fifth reviewer addressed and resolved disagreements on the inclusion of a full-text article.

Data extraction

Four reviewers simultaneously extracted data in duplicate into a predetermined data collection form, and any inconsistencies were settled through consensus or collaboration with a fifth reviewer's input. Data were gathered in the following domains: i) basic study information, such as first author, publication year, and country; ii) study design; iii) follow-up duration and method; and iv) baseline patient demographics, such as the number of participants, median age, gender, and length of stay (LOS) in the hospital and intensive care unit (ICU).

Study quality

The "Joanna Briggs Institute (JBI) Critical Appraisal Checklist for Studies Reporting Prevalence Data" [11] was used to assess the study quality of eligible papers. There are four possibilities for each of the nine items on this checklist. A qualitative assessment of the nine items was done, and we rated each paper's overall quality as excellent, moderate, or low. Two investigators independently assessed the quality, and disagreements were resolved through consensus or collaboration with a third investigator.

Statistical analyses

This meta-analysis was done to assess the Pooled Prevalence (PP) and its 95% confidence interval (CI) of COVID-19's long-term effects at 12 months and beyond. Random-effects or fixed-effects models were used, depending on the heterogeneity of the estimates (I2). Fixed-effects models were applied when studies had I2 ≤ 50%, and if studies showed significant heterogeneity (I2 ≥ 50%), random-effects models were applied. Only symptoms reported by three or more studies were included in the meta-analyses. A funnel plot for prevalence estimates following logit transformation was constructed for each symptom with more than 10 studies to visually detect publication bias. The R studio “version 4.1.2 (2021-11-01)” and the “Meta package (Schwarzer, 2016)” were used to conduct all the analyses. The significance level (0.05) for each two-sided statistical test was set.

Results

**Figure 1:**
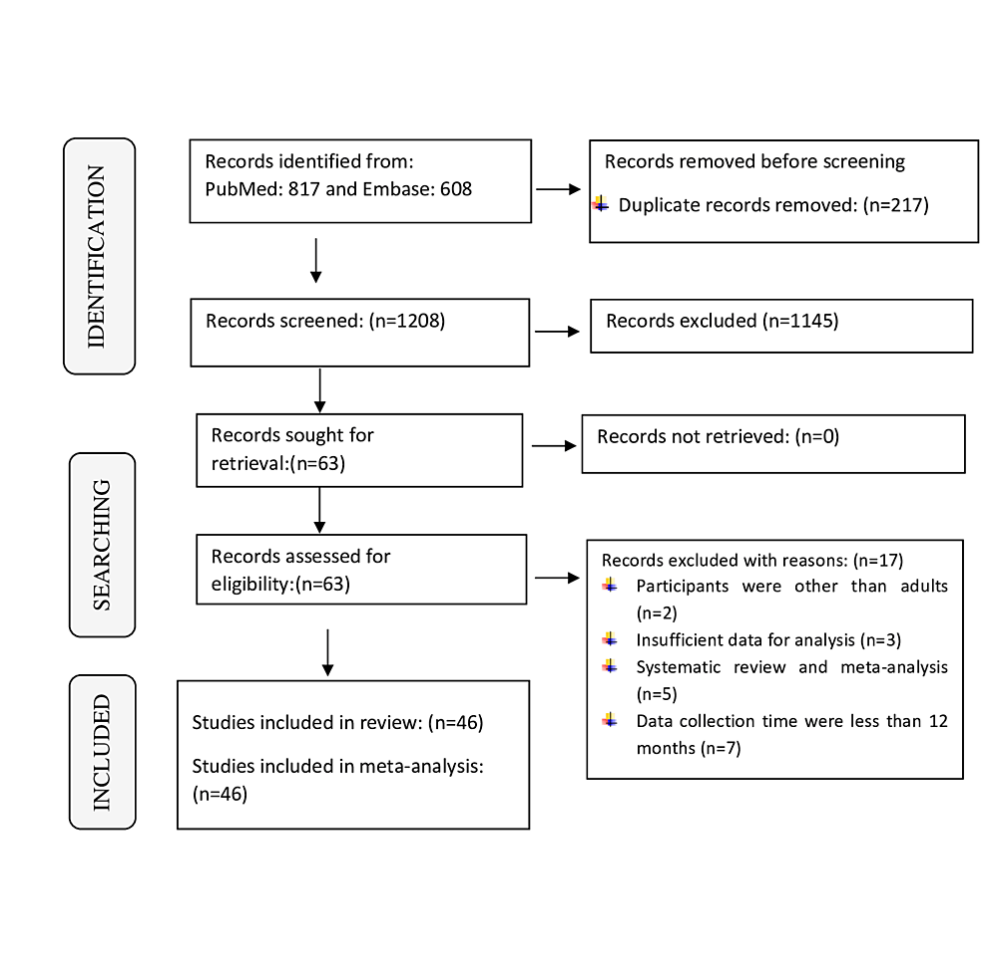
PRISMA flowchart

In total, 1425 records were found during the search of PubMed and Embase. After the abstract and full-text screening, this systematic review and meta-analysis included 46 eligible studies with a total of 17976 COVID-19 survivors. The PRISMA flowchart provided details on the procedure for evaluating the literature (Figure 1).

Table 1 provides a summary of the 46 included studies’ characteristics (12-57). Most of these investigations were conducted in China (n = 13), Italy (n = 11), Spain (n = 7), Germany (n = 3), and Switzerland (n = 2), with a median follow-up length of 685 days from the initial SARS-CoV-2 positive swab being the longest. The number of participants for the listed studies ranges from 51 to 2433. More than half of the included research (n=22) used an in-person method of follow-up, and the preponderance of the included studies (n = 30) were single-center studies.

**Table 1:** Characteristics of included studies F: Female; ICU: Intensive care unit; LOS: Length of stay; M: Male; NR: Not reported; * Data in Median (Inter quartile range); ** Data in mean± Standard deviation

Authors (Year of Publication)	Country	Study setting	Number of participants	Gender M: F	Hospitalization (LOS days)	ICU (LOS days)	Follow-up time	Method of follow-up
Aranda et al. (2022) [12]	Spain	Multicentre	150	87: 63	9.0 (6.0–14.0) *	30.5 (15.8–36.8) *	395 days (384–417) *	In person
Becker et al. (2021) [13]	Switzerland	Bicentric	90	56:34	9.44±6.77 **	15 NR	At 12 Month	NR
Bellan et al. (2021) [14]	Italy	NR	200	122:78	9 (5–16) *	9 (5–16) *	366 days (363–369) *	In person
Boscolo-Rizzo et al. (2021) [15]	Italy	Single centre	304	119:185	Home isolation	Home isolation	At 12 Month	Telephone
Brien et al. (2022) [16]	Ireland	Single centre	61	35:26	13.1(5.8-18.1) *	16(26) NR	430 days (398-458) *	In person
Catalán et al. (2021) [17]	Spain	Single centre	76	67:09	NR	NR	1 year	Telephone
Chai et al. (2021) [18]	China	Multi centre	432	246:252	21 (11–28) *	NR	12.2 months (12.1–12.6) *	Telephone & in person
Fang et al. (2021) [19]	China	Multi centre	1233	591:642	15(10–22) *	NR	363(357–371) * days	NR
Fernández‑de‑las‑Peñas et al. (2021) [20]	Spain	Multi centre	1950	1035:915	11.4 ±11.2**	13 ±14**	11.2±0.5 months **	Telephone
Ferrucci et al. (2022) [21]	Italy	Single centre	53	38:15	11.94 (7.07) *	NR	11.92 ± 1.46 months **	Telephone
Gamberini et al. (2021) [22]	Italy	Multi centre	178	129:49	NR	23 (15–35) *	1Year	Electronic
Huang et al. (2021) [23]	China	Single centre	1276	681:595	14·0 (10·0–20·0) *	18·0 (7·0–30·0) *	12 Months	In person
Huang et al. (2022) [24]	China	Single centre	1192	641:551	14·0 (10·0–20·0) *	18·0 (6·0–30·0) *	685 days (675-698) *	In person
Hussain et al. (2022) [25]	Sweden	Single centre	105	80:25	NR	15 (7.5–26.5) *	365 days (351–379) *	In person
Izquierdo et al. (2022) [26]	Spain	Multi centric	453	260:193	15+13.5 **	48 ±10.6 **	At 12 Month	Telephone
Kim et al. (2022) [27]	Korea	Single centre	241	77:164	NR	NR	454 days (451- 458) *	Telephone
Latronico et al. (2021) [28]	Italy	Single centre	51	NR	NR	NR	At 12 Months	Telephone
Li et al. (2022) [29]	China	Single centre	230	116:114	21 (16–29) *	12 (5.7–9.5) *	At 1 year	In person
Li Z et al. (2022) [30]	China	Single centre	535	216:319	NR	NR	At 1 year	In person
Liao T et al. (2022) [31]	China	Single centre	303	59:244	15 (9–26) *	NR	395 days (382–408) *	In person
Lim RK et al. (2022) [32]	Canada	Single centre	62	27:35	NR	NR	387 days (251–402) *	Telephone
Liu T et al. (2022) [33]	China	Single centre	486	225:261	NR	NR	At 12 months	In person
Liu Y et al. (2022) [34]	China	Single centre	1438	691:747	20 (15-25) *	72 NR	At 12 Months	Telephone
Lombardo MDM et al. (2021) [35]	Italy	Single centre	303	138:165	NR	NR	At 12 Months	Telephone
Maestre-Muñiz MM et al. (2021) [36]	Spain	Single centre	543	275:268	NR	NR	During 1year follow-up	Telephone
Maestrini V et al. (2021) [37]	Italy	Single centre	118	67:51	NR	NR	At 1year follow-up	Telephone
Martino GP et al. (2022) [38]	Italy	Single centre	64	41:23	NR	NR	At 12 months	Telephone
Mazza MG et al. (2022)[39]	Italy	Single centre	192	131:61	NR	NR	387.39 ± 23.67 days**	NR
Noujaim et al. (2022) [40]	France	Multi centre	120	65:55	NR	NR	12 months	In person
Ovrebotten et al. (2022) [41]	Norway	Multi centric	178	105:73	6(3-11) *	NR	389 days (289-462) *	In person
PHOSP COVID collaborative group (2022) [42]	United Kingdom	Multi centre	924	572:319	17.0+24.7 **	NR	13 months (12-13) *	In person
Rank et al. (2021) [43]	Germany	Single centre	83	63:20	NR	NR	At 12 Month	In person
Rass et al. (2021) [44]	Austria	Multi centre	81	48:33	8(0-18) *	31(24-49) *	416 days (401-437) *	In person
Rigoni et al. (2022) [45]	Italy	Single centre	471	301:170	10(6-16) *	NR	12 months	In person & Telephone
Seeble et al. (2021) [46]	Germany	NR	96	53:43	31±32.3 **	NR	12 months	In person
Serra et al. (2022) [47]	Rome	Single centre	109	63:46	15 (9-24) *	NR	12 months	Telephone
Tarraso et al. (2022) [48]	Spain	Multi centre	284	157:127	NR	NR	1 year	in person
Tessitore et al. (2021) [49]	Switzerland	Single centre	184	114:70	11.1 + 9.1 *	NR	1 year + 15 days *	Telephone
Tortajada et al. (2022) [50]	Spain	Single centre	68	49:19	17(15-19) *	NR	12 months	Telephone
Weihe et al. (2022) [51]	Denmark	Single centre	110.	77:33	NR	13.5(8-21) *	12 months	Telephone
Wu et al. (2021) [52]	China	Single centre	83	47:36	29(25-35) *	NR	348 days (341-359) *	In person
Zangrillo et al. (2022) [53]	Italy	Single centre	56	50:06	30(23-44) *	13(9-21) *	349 days (343-356) *	
Zhan et al. (2021) [54]	China	Single centre	121	50:71	24(19-30) *	NR	316 days (311-321) *	In person
Zhang (2021) [55]	China	Multi centre	2433	1205:1228	14(9-20) *	NR	364 days (357-371) *	Telephone
Zhao et al. (2021) [56]	China	Multi centre	94	54:40	15.1 ± 5.71 **	NR	345 days (333- 349) *	In person
Zuschlag (2022) [57]	Germany	Single centre	162	88:74	9.6+9.2 **	NR	1 year	Telephone

**Figure 2:**
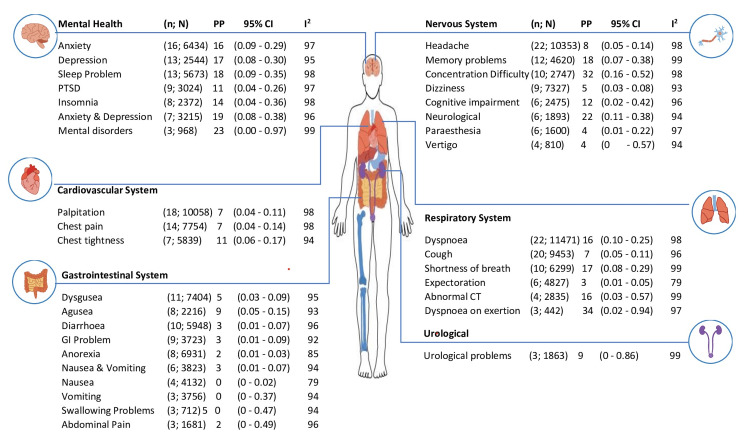
Long COVID-19 symptoms at 12 months and above follow-up periods n:  Number of studies; N: Total number of participants; PP: Pooled prevalence; CI: Confidence interval; I2: Heterogeneity in percentage

**Figure 3:**
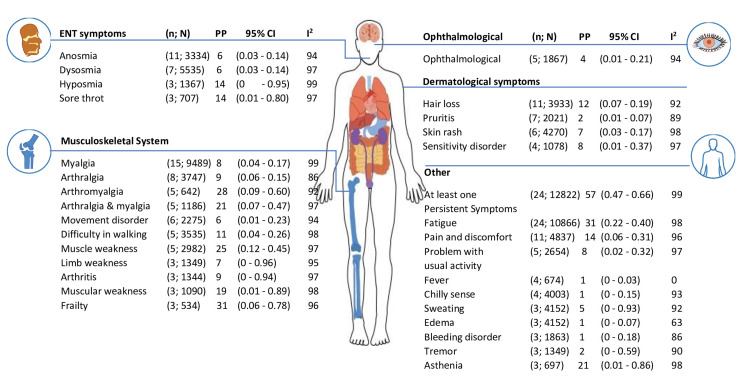
Long COVID-19 symptoms at 12 months and above follow-up periods n:  Number of studies; N: Total number of participants; PP: Pooled prevalence; CI: Confidence interval; I2: Heterogeneity in percentage

In the present review, we found 66 long-term health-related issues associated with COVID-19 (Figures 2, 3), which were reported by a minimum of three studies. The majority of participants experienced several or at least one persistent symptom (57%, 95% confidence interval [CI]: 0.47 to 0.66; n = 24; the number of participants: 12882). The five most prevalent symptoms were: dyspnea on exertion (34%, 95% CI 0.2; 0.94, n = 3, number of participants = 442); difficulty in concentration (32%, 95% CI 0.16; 0.52; n = 10, number of participants = 2747); fatigue (31%, 95% CI 0.22; 0.40, n =24, number of participants = 10866); frailty (31%, 95% CI 0.06; 0.78, n = 3, number of participants = 534); and arthomyalgia (28%, 95% CI 0.09; 0.6, n = 5, number of participants = 642).

In meta-analyses of at least one persistent symptom, including anxiety, depression, fatigue, dyspnea, sleep disturbance, headache, memory problems, chest pain, cough, palpitation, dysgeusia, myalgia, pain, and discomfort, and hair loss (when the number of studies was > 10), there was no indication of publication bias, according to the funnel plots and Egger's tests (p > 0.10).

The study quality of 36 publications was considered good, followed by eight studies of moderate quality and two of low quality. The reasons for the lower quality of these studies include lack of representation (20 single-center studies), small participant numbers, low response rates, the absence of a universal scale for the evaluation of symptoms, and the lack of standardized and trustworthy follow-up techniques, which were sources of possible bias (especially for phone interviews) (Table 2).

**Table 2:** Quality assessment by the Joanna Briggs Institute’s (JBI) critical appraisal tool for prevalence studies

Study	Was the sample frame appropriate to address the target population?	Were study participants sampled in an appropriate way?	Was the sample size adequate?	Were the study subjects and the setting described in detail?	Was the data analysis conducted with sufficient coverage of the identified sample?	Were valid methods used for the identification of the condition?	Was the condition measured in a standard, reliable way for all participants?	Was there appropriate statistical analysis?	Was the response rate adequate, and if not, was the low response rate managed appropriately?	Overall
Aranda et al., 2022 [12]	Yes	Yes	Yes	Yes	Yes	Yes	Yes	Yes	Unclear	High
Becker et al., 2021 [13]	Yes	Yes	Unclear	Yes	Yes	Yes	Yes	Yes	Unclear	Moderate
Bellan et al., 2021 [14]	Unclear	Yes	Unclear	Unclear	Yes	Yes	Yes	Yes	Unclear	Moderate
Boscolo-Rizzo et al., 2021 [15]	Yes	Yes	Yes	No	Yes	Yes	Unclear	Yes	Yes	High
Brien et al., 2022 [16]	Yes	Yes	No	Yes	Yes	Yes	Yes	Yes	Unclear	Moderate
Catalán et al., 2021 [17]	No	Unclear	No	Yes	Yes	Yes	Yes	Yes	Unclear	Low
Chai et al., 2021 [18]	No	No	Yes	Yes	Yes	Yes	Yes	Yes	Yes	Moderate
Fang et al., 2021 [19]	Yes	Yes	Yes	Yes	Yes	Yes	Unclear	Yes	Yes	High
Fernández‑de‑las‑Peñas et al., 2021 [20]	Yes	Yes	Yes	Yes	Yes	Yes	Yes	Yes	Yes	High
Ferrucci et al., 2022 [21]	Unclear	Yes	No	Yes	No	Yes	Yes	Yes	No	Low
Gamberini et al., 2021 [22]	Yes	Yes	Yes	Yes	Yes	Yes	Yes	Yes	Unclear	High
Huang et al., 2021 [23]	Yes	Yes	Yes	Yes	Yes	Yes	Yes	Yes	Yes	High
Huang et al., 2022 [24]	Yes	Yes	Yes	Yes	Yes	Yes	Yes	Yes	Yes	High
Hussain et al., 2022 [25]	Yes	Yes	Yes	Yes	Yes	Yes	Yes	Yes	Unclear	High
Izquierdo et al., 2022 [26]	Yes	Yes	Yes	Yes	Yes	Yes	Yes	Yes	Yes	High
Kim et al., 2022 [27]	Yes	Yes	Yes	Yes	Yes	Yes	Yes	Yes	Unclear	High
Latronico et al., 2021 [28]	No	Yes	No	Yes	Yes	No	Yes	Yes	No	Low
Li et al., 2022 [29]	Yes	Yes	Yes	Yes	Yes	Yes	Yes	Yes	Unclear	High
Li Z et al., 2022 [30]	Yes	Yes	Yes	Yes	Yes	Yes	Yes	No	Unclear	High
Liao T et al., 2022 [31]	Yes	Yes	Yes	Yes	Yes	Yes	Yes	Yes	Yes	High
Lim RK et al., 2022 [32]	No	No	No	No	No	Yes	No	No	Unclear	Low
Liu T et al., 2022 [33]	Yes	Yes	Yes	Yes	Yes	Yes	Yes	Yes	Yes	High
Liu Y et al., 2022 [34]	Yes	Yes	Yes	Yes	Yes	Yes	Yes	Yes	Unclear	High
Lombardo MDM et al., 2021 [35]	Yes	Yes	Yes	Yes	Yes	Yes	Yes	Yes	Unclear	High
Maestre-Muñiz MM et al., 2021 [36]	Yes	Yes	Yes	Yes	Yes	Yes	Yes	Yes	Unclear	High
Maestrini V et al., 2021 [37]	Yes	No	Yes	No	Yes	Yes	Yes	Yes	Unclear	Moderate
Martino GP et al., 2022 [38]	No	No	No	Yes	Yes	Yes	Yes	Unclear	Unclear	Low
Mazza MG et al., 2022 [39]	Yes	Yes	Yes	Yes	Yes	Yes	Yes	Yes	Yes	High
Noujaim et al., 2022 [40]	Yes	Yes	Yes	Yes	Yes	Yes	Yes	Yes	Unclear	High
Ovrebotten et al., 2022 [41]	No	Yes	Yes	Yes	Yes	Yes	Yes	Yes	Unclear	Moderate
PHOSP COVID collaborative group, 2022 [42]	Yes	Yes	Yes	Yes	Yes	Yes	Yes	Yes	Yes	High
Rank et al., 2021 [43]	No	No	No	Yes	Yes	Yes	Yes	Yes	No	Low
Rass et al., 2021 [44]	Unclear	Unclear	No	Yes	No	Unclear	Yes	Yes	Unclear	Low
Rigoni et al., 2022 [45]	Yes	Yes	Yes	Yes	Yes	Yes	Yes	Yes	Yes	High
Seeble et al., 2021 [46]	Yes	Unclear	No	Yes	Unclear	Yes	Yes	Yes	Unclear	Moderate
Serra et al., 2022 [47]	Yes	Unclear	No	No	Yes	Yes	Yes	Yes	Unclear	Low
Tarraso et al., 2022 [48]	No	Yes	Yes	Yes	Yes	Yes	Yes	Yes	Yes	High
Tessitore et al., 2021 [49]	Yes	Yes	Yes	Yes	Yes	Yes	Yes	Yes	Yes	High
Tortajada et al., 2022 [50]	Yes	Yes	No	Yes	Unclear	Yes	Yes	Yes	Yes	High
Weihe et al., 2022 [51]	Yes	Yes	Yes	Yes	Yes	Unclear	Yes	Unclear	Unclear	Moderate
Wu et al., 2021 [52]	No	No	No	Yes	Yes	Yes	Yes	Yes	Yes	Moderate
Zangrillo et al., 2022 [53]	No	No	No	Yes	Yes	Yes	Yes	Yes	Yes	Moderate
Zhan et al., 2021 [54]	No	No	No	No	Yes	Yes	Yes	No	No	Low
Zhang et al., 2021 [55]	Yes	Yes	Yes	Yes	Yes	Yes	Yes	Yes	Yes	High
Zhao et al., 2021 [56]	Yes	Yes	No	Yes	Yes	Yes	Yes	Yes	Unclear	Moderate
Zuschlag et al., 2022 [57]	Yes	Yes	Yes	Yes	Yes	Yes	Yes	Yes	Unclear	High

Discussion

This systematic review and meta-analysis contribute to the sparse literature available on the health impacts of post-COVID-19 disease, particularly sudden cardiac death, prolonged arthralgia, neurological problems, and autoimmune diseases. There is a very limited amount of information available on such impacts, and none of them have evaluated these symptoms for as long as 12 months, to the best of our knowledge. Developing effective interventions, treatments, and vaccine strategies requires a comprehension of the long-term effects of COVID-19. In earlier research, the COVID-19 effects were examined after three months, six months, nine months, or longer.

The studies that investigated the health effects of adult patients who had recovered from COVID-19 and were included in our study had the longest follow-up periods. In this review, we presented the pooled prevalence (PP) of long-term effects of COVID-19 at 12 months and above based on a meta-analysis of 46 investigations encompassing 17976 cases of COVID-19. These findings suggested that COVID-19 patients may experience a range of short-, mid-, and long-term consequences even if they fully recover.

At the 12-month follow-up, 57% of patients exhibited at least one symptom. The results were consistent with research that indicated 53% of patients had at least one symptom after 12 months of COVID-19 infection [15]. In Lombardo's study [35], the proportion was larger (over 80%), whereas in, Bellan et al.’s [14] study, a smaller proportion (about 40%) was reported. The inconsistent findings of the 12-month follow-up studies show that additional original research is required to determine the long-term effects of COVID-19. This suggests that COVID-19 may have long-lasting effects on organs.

We report a prevalence of fatigue of 31% at 12 months or more, which is comparable to Michelen et al.’s [8] findings (30.1%), but lower than the studies reported by Iqbal et al. [58] (37%), and the meta-analysis by Alkodaymi et al. [59] (41%). It has already been noted that different viral and bacterial infections can cause persistent fatigue that lasts for up to six months, but the processes causing this symptom are yet unknown. These might be brought on by changes in immune system functioning, which have been linked to potential post-viral fatigue [60].

In terms of persistent dyspnea, Fernandez de las Peas et al. [20] (23.3%) and Huang et al. [24] (30%) reported lower percentages than we did (34%), although Aranda et al. [12] (62%), and Gamberini et al. (58.4%), reported a greater prevalence of dyspnea. This phenomenon may be explained by the mixed population of critical and non-critical COVID-19 patients or by the fact that both hospitalized and out-of-hospital patients were included in the study's population [22].

This is also true for arthromyalgia, with Gamberini et al. [22] (34.8% prevalence) and Catalán et al. [17] (37.5% prevalence) reporting a higher prevalence than this meta-analysis (28%). This discrepancy may be due to the subjective nature of this symptom, population differences, and the use of different assessment scales.

The greatest strategy to lessen the effects of COVID-19 is to prevent infection, for which vaccination is crucial because the disease's long-term implications are still unknown. Additionally, enhancing COVID-19 testing can aid in the earliest possible diagnosis and management. Furthermore, studies can be conducted on the use of artificial intelligence in all areas of COVID-19 [61].

Limitation and strength

To date, our work is one of the largest and most comprehensive systematic reviews of symptoms that persist after an acute COVID-19 infection. The included studies and their designs' inherent limitations, however, are present in the study. First, the literature we reviewed lacked unified terminology, standardized recording methods, and the categorization of various symptoms under general headings. We were unable to compare the incidence and prevalence of these symptoms between studies as a result. Second, numerous studies did not describe the severity of the disease, and the results were only given to the entire cohort. This results in incorrect estimations of symptom frequencies when all symptoms of different disease severity are combined. Lastly, the significant statistical heterogeneity constrains the interpretation of the pooled frequencies.

## Conclusions

This comprehensive review and meta-analysis showed that a significant fraction of COVID-19 survivors experience a variety of physical, cognitive, and behavioral health problems that last for at least a year. Understanding the underlying pathophysiologic mechanisms and conducting intervention studies to treat or stop the persistence of these long-term consequences are urgently needed. However, the studies available on long-term COVID-19 are extremely diverse. Future research must include comparators, standardized symptom criteria, and an extended follow-up period.
